# Supplementary material for: Relevance of Host Cell Surface Glycan Structure for Cell Specificity of Influenza A Viruses
Source: Viruses. 2023 Jul 5;15(7):1507. doi: 10.3390/v15071507 (PMC10385328; doi:10.3390/v15071507)
Supplement: Supplementary file 1 [file viruses-15-01507-s001.zip › viruses-2416578-supplementary.pdf]

Supporting Information

Relevance of host cell surface glycan structure for cell specificity of influenza A viruses

Markus Kastner [1,11,*], Andreas Karner [1,11,*], Rong Zhu [1,*], Qiang Huang [2], Andreas Geissner [3,4,11], Anne Sadewasser [5,11], Markus Lesch [6], Xenia Wörmann[6], Alexander Karlas[6,11], Peter H. Seeberger [3,4], Thorsten Wolff [5], Peter Hinterdorfer [1], Andreas Herrmann[7] and Christian Sieben [8,9,§]

[1] *Institute for Biophysics, Johannes Kepler University Linz, 4020 Linz, Austria*

[2] *State Key Laboratory of Genetic Engineering, Shanghai Engineering Research Center of Industrial Microorganisms, MOE Engineering Research Center of Gene Technology, School of Life Sciences, Fudan University, Shanghai 200438, China*

[3] *Department for Biomolecular Systems, Max Planck Institute for Colloids and Interfaces, 14476 Potsdam, Germany*

[4] *Institute of Chemistry and Biochemistry, Freie Universität Berlin, 14195 Berlin Germany*

[5] *Div. of Influenza and other Respiratory Viruses, Robert Koch-Institute, 13353 Berlin, Germany*

[6] *Molecular Biology Department, Max Planck Institute for Infection Biology, 10117 Berlin, Germany*

[7] *Institut für Chemie und Biochemie, Freie Universität Berlin, Altensteinstraße 23a, 14195 Berlin Germany*

[8] *Nanoscale Infection Biology Group, Department of Cell Biology, Helmholtz Centre for Infection Research, 38124 Braunschweig, Germany,*

[9] *Institute for Genetics, Technische Universität Braunschweig, 38106 Braunschweig, Germany*

[11] *Current address: M.K.: Materials Characterization Lab (MCL), Materials Research Institute (MRI), Pennsylvania State University, University Park, PA 16802, USA; A.K.: University of Applied Sciences Upper Austria, School of Medical Engineering and Applied Social Sciences, Garnisonstr. 21, 4020 Linz, Austria; A.G.: Department of Chemistry, University of British Columbia, 2036 Main Mall, Vancouver BC, Canada V6T 1Z1; A.S.: Secarna Pharmaceuticals GmbH & Co. KG, Am Klopferspitz 19, 82152 Planegg/Martinsried, Germany; A.K.: Viral Vectors and Gene Therapeutics, ProBioGen AG, 13086 Berlin, Germany*

[*] These authors contributed equally to this work

[§] Corresponding author: christian.sieben@helmholtz-hzi.de (C.S.)

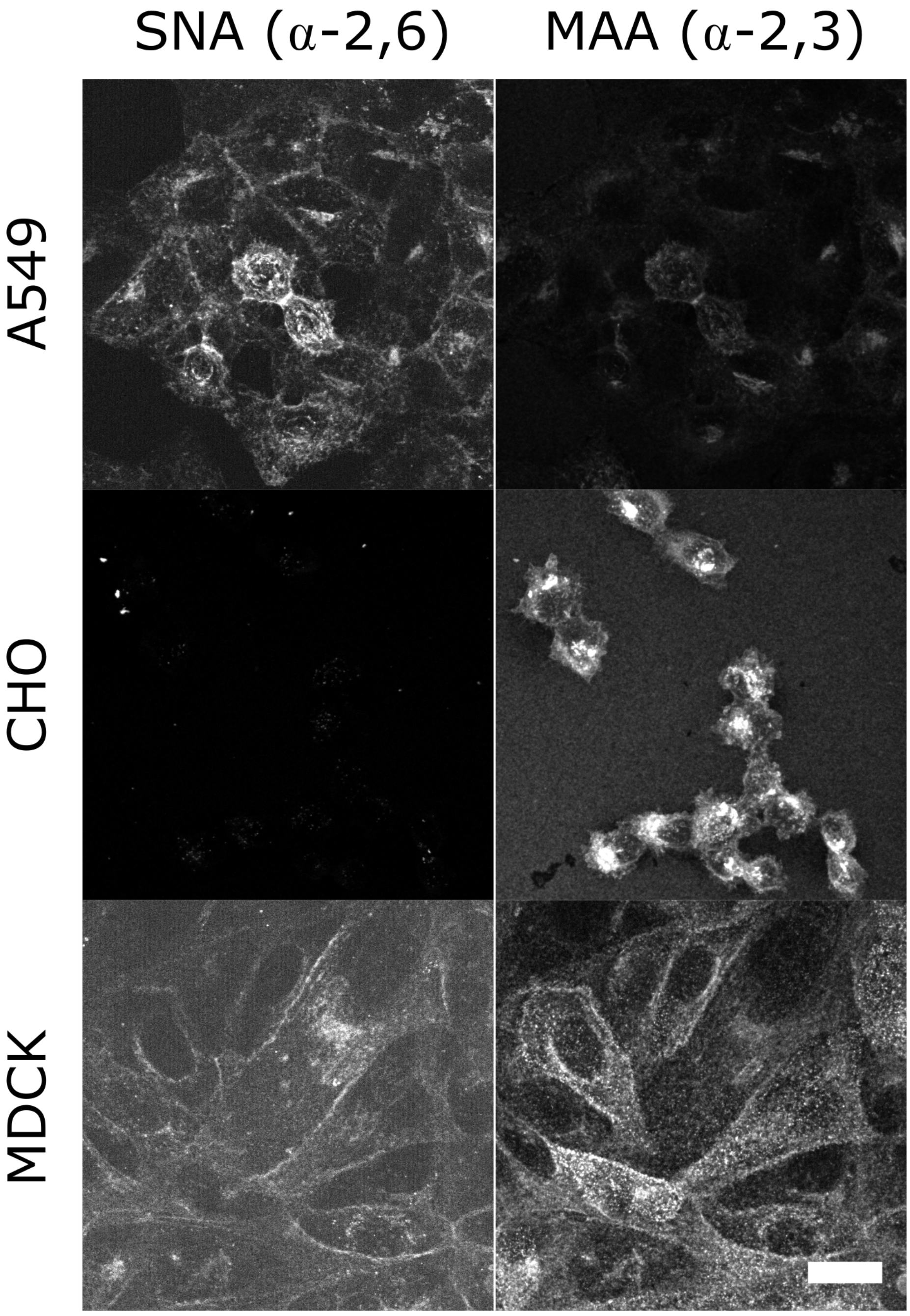

Supplementary Figure S1. Surface sialic acid characterization by fluorescence confocal microscopy. MDCK, CHO, and A549 cells were washed with PBS and fixed with PFA for 20 min at room temperature. The cells were incubated with SNA–FITC (10 μg/ml) and MAA–Alexa647 (50 μg/ml) for 20 min, washed with PBS and imaged by confocal microscopy.

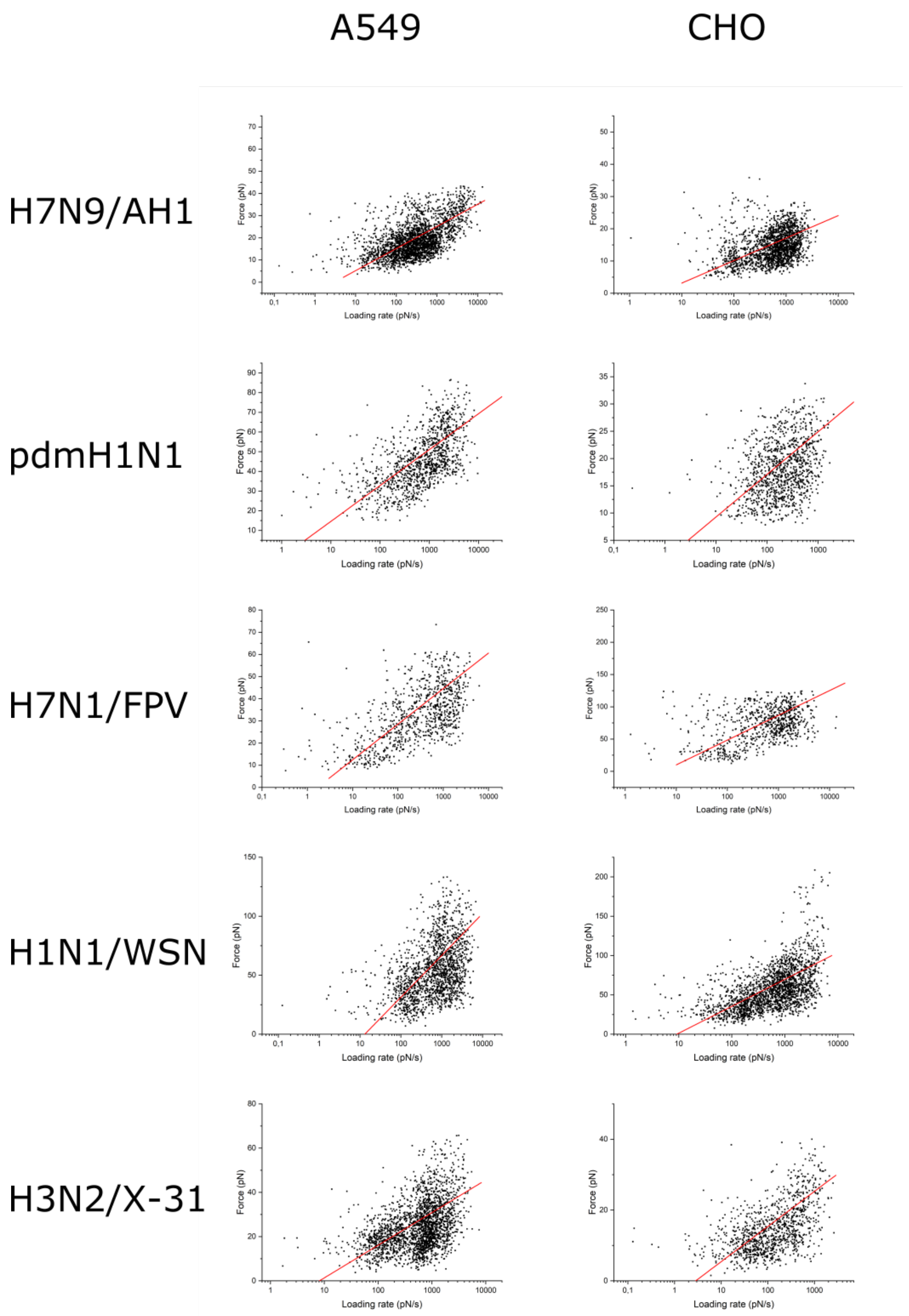

Supplementary Figure S2. Overview showing force vs. loading rate scatter plots for all virus-cell combinations. Scatter plots showing unbinding force F plotted against the loading rate r of every individual force curve. From those data, the values for k_{off} and x_u were determined (**Table 2**). For each virus-cell combination, we measured between 12-15 cells.

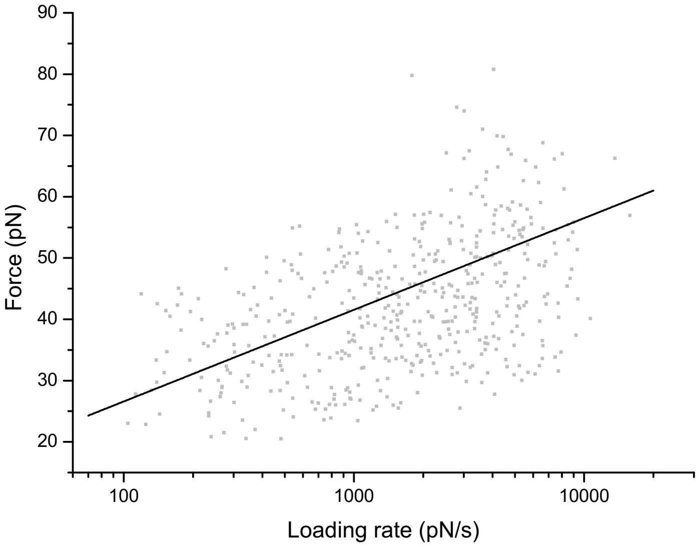

Supplementary Figure S3. SVFS dynamic force spectra of H7N9 AH1 interacting with single receptors on living MDCK cells. Scatter plot showing unbinding force F plotted against the loading rate r of every individual force curve. From those data, the values for k_{off} and x_u were determined to be 0.256 +/- 0.00169 s^{-1} and 6.160 +/- 0.0146 Å (mean ± SD), respectively. We measured between 12-15 cells.

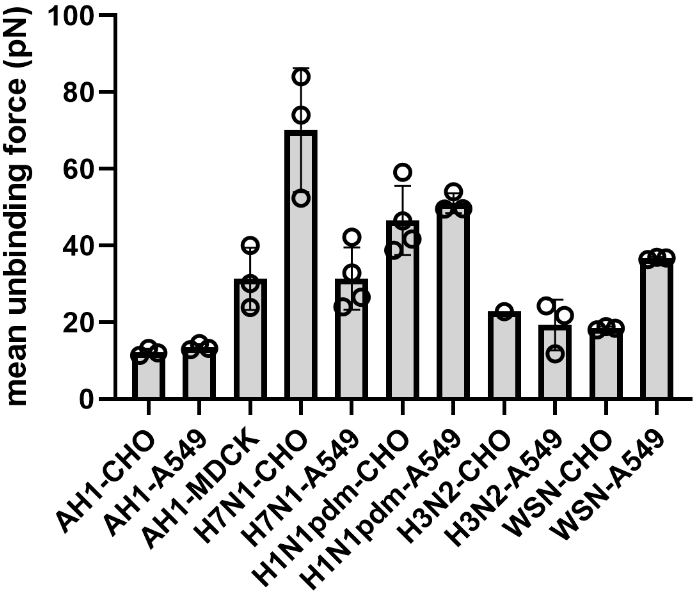

Supplementary Figure S4. Comparison of the mean unbinding force at pulling velocity 4 µm/sec. Each point represents one cell of the shown virus-cell interaction pair. Shown are mean ± SD, n=3.

Supplementary Table S1. Comparison of SVFS results obtained by using two different fitting approaches. Loading rates *LR* can either be obtained by using the mean effective spring constant <k_{eff}> for each pulling velocity *v* (LR = v*k_{eff}) or, using a more adapted approach reported previously and now used in this study, calculated for each individual force-distance curve. Each fitting approach results in slightly different fitting parameters. Dissociation rate k_{off}, separation from the energy barrier x_u, and average bond lifetime τ_{off} obtained by fitting the SVFS data to a single energy barrier binding model.

New Analysis, MLE from force vs. loading rate scatter plots (mean ± SD)

Virus	Cell	k_off (1/s)		x_beta (A)		tau (s)	
H1N1_WSN	A549	0.851	0.0525	2.67	0.0445	1.1751	0.0725
H1N1_WSN	CHO	0.62	0.0355	2.77	0.0397	1.6129	0.0924
H3N2_X31	A549	1.27	0.0704	6.42	0.0897	0.7874	0.0436
H3N2_X31	CHO	0.66	0.0472	9.54	0.183	1.5152	0.1084
H3N2_X31	MDCK	1.61	0.141	9.11	0.198	0.6211	0.0544

Old Analysis, fit with grouped loading rates, (mean ± SD)

Virus	Cell	k_off (1/s)		x_beta (A)		tau (s)	
H1N1_WSN	A549	1.22	0.32	1.8	0.3	0.8197	0.2150
H1N1_WSN	CHO	1.16	0.13	2.4	0.3	0.8621	0.0966
H3N2_X31	A549	0.64	0.52	4.2	4.1	1.5625	1.2695
H3N2_X31	CHO	0.18	0.17	26.1	31.1	5.5556	5.2469
H3N2_X31	MDCK	0.45	0.35	8.4	7.9	2.2222	1.7284
